# A Study on the Dynamic Forming Mechanism Development of the Negative Poisson’s Ratio Elastomer Molds—Plate to Plate (P2P) Forming Process

**DOI:** 10.3390/polym13193255

**Published:** 2021-09-24

**Authors:** Yung-Jin Weng, Jen-Ching Huang, Yueh-Yang Chen, Shao-Teng Hsu, Zu-Rong Zhang

**Affiliations:** 1 Department of Mechanical and Energy Engineering, National Chiayi University, Chiayi 60004, Taiwan; love89783@gmail.com (Y.-Y.C.); s1053121@mail.ncyu.edu.tw (S.-T.H.); s1053144@mail.ncyu.edu.tw (Z.-R.Z.); 2 Department of Mechanical Engineering, Tungnan University, New Taipei City 222, Taiwan; jc-huang@mail.tnu.edu.tw

**Keywords:** negative Poisson’s ratio, elastomer, microstructure, dynamic forming, plate to plate (P2P)

## Abstract

This study proposed a dynamic forming mechanism development of the negative Poisson’s ratio elastomer molds—plate to plate (P2P) forming process. To dynamically stretch molds and control the microstructural shape, the proposal is committed to using the NPR structure as a regulatory mechanism. The NPR structural and dynamic parallel NPR-molds to control microstructure mold-cores were simulated and analyzed. ANSYS and MATLAB were used to simulate and predict dynamic NPR embossing replication. The hot-embossing and UV-curing dynamic NPR P2P-forming systems are designed and developed for verification. The results illustrated that the dynamic forming mechanism of the negative Poisson’s ratio elastomer molds proposed by this study can effectively control microstructure molds. This can effectively predict and calculate the geometrical characteristics of the microstructures after embossing. The multi-directional dynamic NPR microstructural replication process can accurately transfer microstructures and provide high transfer rate-replication characteristics.

## 1. Introduction

With the development of microstructure technology, various microstructural components with different shapes, multiple directions, or even curved surfaces are now needed as light-guided components, electrical materials, and biomedical modules. This is to meet the needs of academic research and of the high-tech industry. Stephen Y. Chou [1,2] et al. proposed a new micro/nano-lithography process with the advantages of fast production and low cost in 1996. It made a structure with a high-resolution line-width below 50 nm. It started the subsequent process innovations. In recent years, Chang and Yang et al. [3] improved the shortcoming of direct embossing in the traditional hot-embossing machine. They developed a fluid embossing method to replicate microstructures and replicated microstructural patterns on large-area wafers by the isotropic and isobaric characteristics of the fluid. Lee et al. [4] proposed a liquid roll-to-roll imprint technology (R-LTIL) with a large-area process and both a micron-scale and nano-scale hybrid structure. It can transfer structures with a pore diameter of 350 nm and a height of 250 nm onto 6 inch silicon wafers and micron microlens substrates. Huichun et al. [5] proposed a roll-to-roll (R2R) embossing process in 2018; it became a simple and rapid method to prepare semi-ellipsoidal microlens arrays (SEMA). The replication error of transferring the polydimethylsiloxane (PDMS) mold from a preformed SEMA prototype is less than 1.59%. Aaron et al. [6] developed a method to accurately culture cells with PDMS. Moreover, the micro/nano-structural substrates of different sizes and shapes [7,8] prepared by different processing methods (top-down, bottom-up) have high SERS enhancement factors and can be used for medical detection. At present, microstructural components, such as micro-nano gratings [9,10,11,12,13,14], polarizers, and lenses, can be completed by technologies such as nano-imprint, exposure, and development [15,16,17,18,19,20,21,22,23,24,25,26,27,28,29,30,31,32,33,34,35]. However, there is a problem of material selection if etching is used for microstructure fabrication. According to previous stumolds, in the etching process, some materials cannot be used to fabricate the microstructural patterns according to the design as required by researchers (the problem of material selection). If special complementary structures can be prefabricated with elastic molds, there will be more choices in the replication process. It has been previously researched that the construction of the mold mechanical model, specifically the Bayesian inversion, is a probabilistic model to identify the material parameters in mechanical systems. Noii, N. et al. [36] developed a step-wise Bayesian inversion framework for ductile fractures to provide accurate knowledge regarding the effective mechanical parameters. Khodadadian, A et al. [37] proposed a Bayesian approach to estimate material parameters for propagating fractures in elastic solids as well as to solve the problem on a relatively coarse mesh and fit the parameters. For example, in the Fresnel lens processing within the elastic limit, the molds with adjustable Poisson’s ratios are stretched and formed by controlling the shape and characteristic parameters of microstructural components through multi-axial stretching. It is estimated that various types of Fresnel lenses with single microstructural components can be obtained. The Fresnel lenses with the best spotlight effect and efficiency can be made quickly and easily. Therefore, to meet the needs of practical applications, the microstructural characteristics and mold sizes can be adjusted slightly and stably by the dynamic stretching according to the adjustable Poisson’s ratio. This is helpful and innovative for industrial applications.

This study proposes the dynamic forming mechanism development of the negative Poisson’s ratio elastomer molds—plate to plate (P2P) forming process. In this study, funnel-shaped and SIN wave-shaped molds with NPR structures and flexible NPR structures are fabricated with both a thermoplastic elastomer (TPE) and PDMS for the mechanical property test (creep and stress relaxation). Through numerical simulation, the NPR geometric position changes and the stress distribution of molds in the uniaxial dynamic stretching are analyzed. This is conducted in order to adjust the different line-width of NPR structures and to build a mechanical model of the flexible NPR microstructure molds. A stretching test is carried out to obtain the predictable micro-deformation range of the linear elasticity. The microstructure shape is fine-tuned by the self-developed fixture mechanism, which can fine-tune the dynamic NPR mold control system. In addition, key processes such as (1) the uniaxial hot-embossing NPR microstructure replication system and (2) the uniaxial UV-curing NPR microstructure replication system are developed. It is anticipated that through a series of stumolds, a prediction model on the viscoelasticity response of the NPR structure and the material can be both integrated and summarized for simulation evaluation and experimental analysis, establishing the mechanical modeling and dynamic axial forming mechanisms of negative Poisson’s ratio polymer molds.

## 2. Stretching Deformation Mechanism of Negative Poisson’s Ratio Elastomer Molds

### 2.1. Prediction Modeling and Computational Evaluation Method on the Viscoelasticity Response of the NPR Structure and Material

In this study, NPR polymer molds are controlled by the axial stretching method within the elastic range of core materials. Therefore, under fixed stretching force (stretching stress) and the fixed strain effect, there will be opportunities for creep and stress relaxation over the stretching time. These are expected to build a mechanical model of the negative Poisson’s ratio polymer molds. In the dynamic forming process, the real-time prediction and effective forming process can be obtained. The exact geometric dimension changes of microstructure molds can be learned at any time in the dynamic control process. To construct a mechanical model of negative Poisson’s ratio core materials, dampers and springs are used as the basic components. The Maxwell model and Kelvin–Voigt model are adopted as methods to construct and evaluate the mechanical properties of the dynamic stretching of a series of molds, as well as to obtain more accurate evaluations. This study has developed a multi-component model prediction architecture to describe the NPR viscoelasticity mechanical model designed herein, based on the Maxwell and Kelvin–Voigt methods.

### 2.2. Prediction Method for the Uniaxial Stretching Microstructure Position with the Dynamic NPR Mold Control System

**Figure 1:**
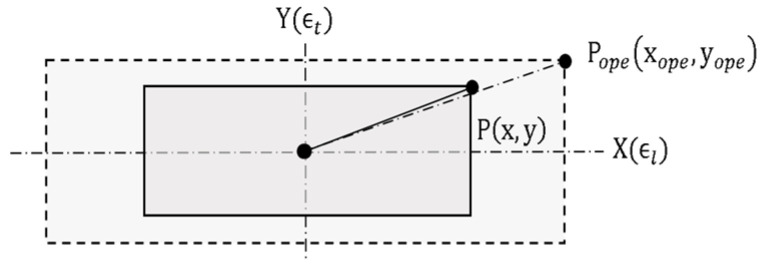
Dynamical stretching geometry of the uniaxial flexible NPR microstructure mold.

In this section, the uniaxial NPR is used in dynamic mold control to discuss the geometric position of negative Poisson’s ratio stretching. Here, P(x,y) is the original geometric position of the flexible mold structure and $P_{ope}\left(x_{ope},y_{ope}\right)$ is the final geometric position after dynamic deformation. According to Figure 1, the equation of the stretching microstructure position is designed and deduced, as shown in Equation (1).

(1) $$\vec{P_{ope}}=\overset{↔}{M_{ope}}\;\cdot \;\vec{P}{\left(\begin{array}{c} x\\ y\\ z \end{array}\right)}_{ope}\approx \;\left(\begin{array}{ccc} 1+\epsilon_{l} & 0 & 0\\ 0 & 1+\epsilon_{t} & 0\\ 0 & 0 & 1 \end{array}\right)\left(\begin{array}{c} x\\ y\\ z \end{array}\right)$$

where, $\epsilon_{l}$ correspond to the longitudinal strain and $\epsilon_{t}$ corresponds to the transverse strain.

### 2.3. Effects of Multidirectional Stretching on Molds under External Force

**Figure 2:**
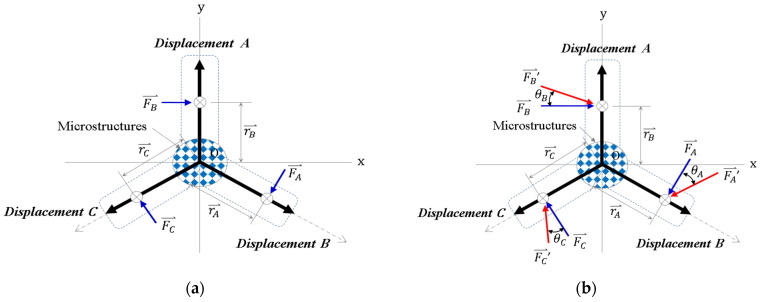
Effects of multi-directional stretching on the mold under external force: (**a**) forward external thrust and (**b**) angled external thrust.

Environmental parameters and machine stability are required to be adjusted during the process of dynamic mold stretching and control. Uncertain external thrust (non-uniform material, mold inclination, and other factors) may cause failures during forming. The external thrust in the three-directional mold stretch forming process may affect molds, as shown in Figure 2.

The effect of the forward external thrust on the total torque (Equation (2)) of a mold is as follows:

(2) $$\begin{array}{c} \sum \left(\vec{r}\times \vec{F}\right)={\vec{r}}_{A}\times {\vec{F}}_{A}+{\vec{r}}_{B}\times {\vec{F}}_{B}+{\vec{r}}_{C}\times {\vec{F}}_{C}\\ \left(M_{R}\right)=\left|\begin{array}{ccc} \vec{i} & \vec{j} & \vec{k}\\ r_{Ax} & r_{Ay} & 0\\ F_{Ax} & F_{Ay} & 0 \end{array}\right|+\left|\begin{array}{ccc} \vec{i} & \vec{j} & \vec{k}\\ r_{Bx} & r_{By} & 0\\ F_{Bx} & F_{By} & 0 \end{array}\right|+\left|\begin{array}{ccc} \vec{i} & \vec{j} & \vec{k}\\ r_{Cx} & r_{Cy} & 0\\ F_{Cx} & F_{Cy} & 0 \end{array}\right|\\ =\left[\left(F_{Ay}r_{Ax}-F_{Ax}r_{Ay}\right)\vec{k}+\left(F_{By}r_{Bx}-F_{Bx}r_{By}\right)\vec{k}+\left(F_{Cy}r_{Cx}-F_{Cx}r_{Cy}\right)\vec{k}\right]\\ =\left[\left(F_{Ay}r_{Ax}+F_{By}r_{Bx}+F_{Cy}r_{Cx}\right)-\left(F_{Ax}r_{Ay}+F_{Bx}r_{By}+F_{Cx}r_{Cy}\right)\right]\vec{k} \end{array}$$

The effect of the angled external thrust on the total torque of a mold is shown in Equation (3). It shall be considered in the process of embossing replication to avoid failures during forming.

(3) $$\begin{array}{c} \sum \left(\vec{r}\times \vec{F^{\prime }}\right)={\vec{r}}_{A}\times {{\vec{F}}_{A}}^{\prime }+{\vec{r}}_{B}\times {{\vec{F}}_{B}}^{\prime }+{\vec{r}}_{C}\times {{\vec{F}}_{C}}^{\prime }\\ \left(M_{R}\right)=\left|\begin{array}{ccc} \vec{i} & \vec{j} & \vec{k}\\ r_{Ax} & r_{Ay} & 0\\ {F_{Ax}}^{\prime } & {F_{Ay}}^{\prime } & {F_{Az}}^{\prime } \end{array}\right|+\left|\begin{array}{ccc} \vec{i} & \vec{j} & \vec{k}\\ r_{Bx} & r_{By} & 0\\ {F_{Bx}}^{\prime } & {F_{By}}^{\prime } & {F_{Bz}}^{\prime } \end{array}\right|+\left|\begin{array}{ccc} \vec{i} & \vec{j} & \vec{k}\\ r_{Cx} & r_{Cy} & 0\\ {F_{Cx}}^{\prime } & {F_{Cy}}^{\prime } & {F_{Cz}}^{\prime } \end{array}\right|\\ =[(r_{Ay}{F_{Az}}^{\prime }\vec{i}+r_{Ay}{F_{Ay}}^{\prime }\vec{k}-r_{Ay}{F_{Ax}}^{\prime }\vec{k}-r_{Ax}{F_{Ay}}^{\prime }\vec{j})\\ +(r_{By}{F_{Bz}}^{\prime }\vec{i}+r_{Bx}{F_{By}}^{\prime }\vec{k}-r_{By}{F_{Bx}}^{\prime }\vec{k}-r_{Bx}{F_{By}}^{\prime }\vec{j})\\ +(r_{Cy}{F_{Cz}}^{\prime }\vec{i}+r_{Cx}{F_{Cy}}^{\prime }\vec{k}-r_{Cy}{F_{Cx}}^{\prime }\vec{k}-r_{Cx}{F_{Cy}}^{\prime }\vec{j})] \end{array}$$

## 3. Experimental

### 3.1. Geometry Design of NPR Structure, Bonding in Mold Preparation, and Flexible NPR Microstructure Mold Preparation

#### 3.1.1. Geometry Design of NPR Structure and Bonding in Mold Preparation

**Figure 3:**
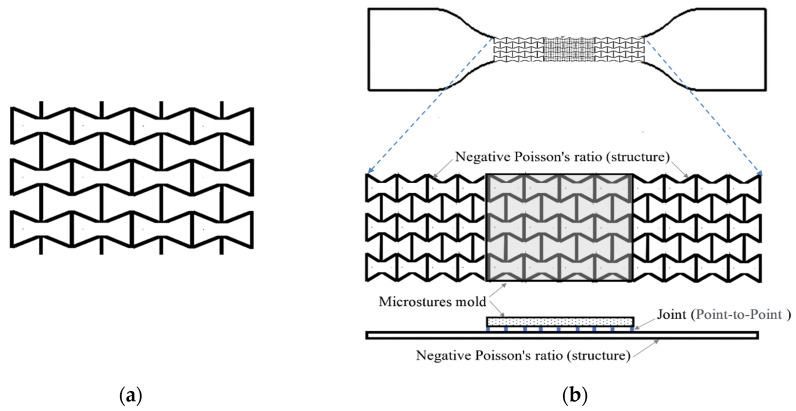
With a flexible NPR structure: (**a**) funnel-shaped and (**b**) microstructure mold-bonding in preparation.

In this section, the design and fabrication of the funnel-shaped flexible NPR microstructure molds are discussed. Through numerical simulation, the NPR geometric position changes and the stress distribution of molds in uniaxial dynamic stretching were analyzed. The stretching, creep, and stress relaxation tests were carried out to test the mechanical properties of the constructed NPR microstructure molds. These tests allowed us to obtain the mechanical properties and deformation within the elastic range according to the standard sample specifications, as well as to build a mechanical model of the flexible NPR microstructure molds. In this study, the main characteristic parameters (angle and line width) were obtained based on the geometry of funnel-shaped flexible NPR microstructures. These are required for the design of the mechanical properties and Poisson’s ratio changes of different scale parameters, as shown in Figure 3a. The Poisson’s ratio changes during stretching within the elastic deformation range were simulated by ANSYS. The uniaxial parallel flexible NPR microstructure mold bonding was adopted (Figure 3b). 

The point-to-point array parallel connection at the intersection of NPR structures was purposed with connecting PDMS female molds in parallel to form an NPR structure mold layer which is divided into lower NPR structure layers and upper layers of PDMS microstructure female molds. PDMS or TPE can be used as the material for the structural layer. The contact faces of two layers were cross-linked by PDMS based on the cross-linking characteristics of polymer materials. Due to the microstructures on PDMS female molds, the lower NPR structure layer was controlled by dynamic stretching. The microstructures on the upper layer of PDMS female molds would expand with the negative Poisson’s ratio changes of NPR structures on the lower layer. This dynamically controls the deformation of the microstructure dimensions within the elastic range.

In this experiment, elastic flexible polydimethylsiloxane was used as the mold material mainly because of its flexible characteristics. The mold material used by our team is also known as organic polymeric material, which is non-toxic, non-flammable, and highly elastic. It has been previously reported that the PDMS material of the mold, which are the refractive indexes for each mold, are related to different variations of the synthesis parameters and curing temperature. In this experiment (refractive indexes = 1.43), we used uniform synthesis parameters (10:1) and the same curing temperature (140 °C), therefore there were no disconnecting aspects in relation to this.

#### 3.1.2. Uniaxial Parallel Flexible NPR Microstructure Mold Preparation

**Figure 4:**
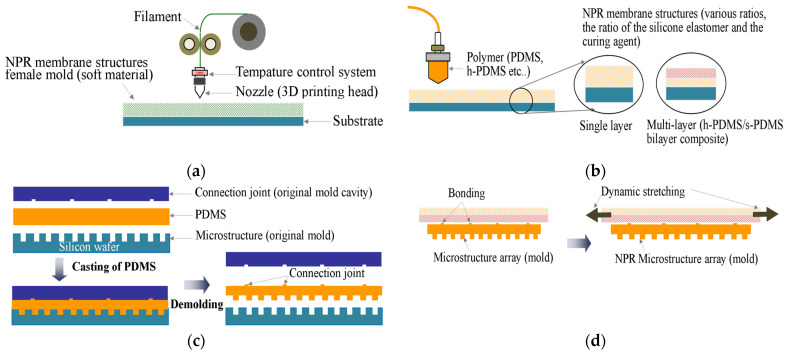
The procedure to fabricate uniaxial parallel flexible NPR microstructure molds was as follows: (**a**) the complementary female molds of NPR structures were fabricated by high-precision 3D printing technology; (**b**) PDMS or h-PDMS/s-PDMS bilayer composites were accurately injected into the complementary cavity of NPR membrane structures to cure and form NPR structures; (**c**) PDMS microstructure molds with fixed bonding points at the top and microstructural arrays at the bottom were prepared; and (**d**) the fixed connections at the top of PDMS microstructure molds were bonded to NPR structures to obtain the point-bonded NPR PDMS microstructure molds after curing.

The procedure to fabricate uniaxial parallel flexible NPR microstructure molds was as follows (Figure 4): (a) the complementary female molds of NPR structures defined in this study were fabricated by high-precision 3D printing technology; (b) PDMS or h-PDMS/s-PDMS bilayer composites defined in this study were accurately injected into the complementary cavity of NPR membrane structures to cure and form NPR structures; (c) PDMS microstructure molds with fixed bonding points at the top and microstructural arrays at the bottom were prepared; and (d) the fixed connections at the top of PDMS microstructure molds were bonded to NPR structures to obtain the point-bonded NPR PDMS microstructure molds after curing.

### 3.2. Dynamic NPR P2P-Forming System Development and Uniaxial Parallel NPR-Embossing Steps

#### 3.2.1. Dynamic NPR P2P-Forming System Design

**Figure 5:**
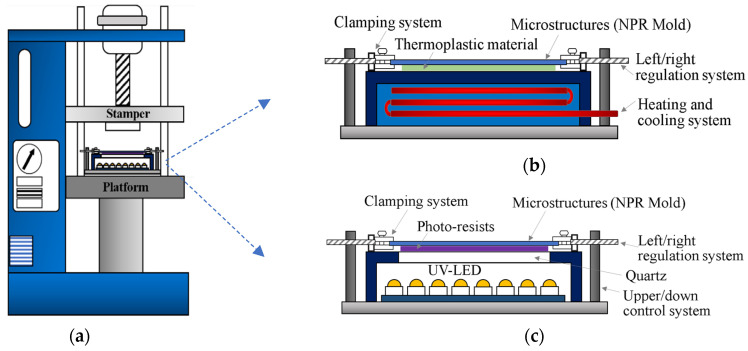
P2P-forming system for the uniaxial NPR microstructure replication (**a**) embossing system and mold system; (**b**) uniaxial NPR microstructure hot-embossing replication system; and (**c**) uniaxial NPR microstructure UV-curing replication system.

In this study, the existing embossing system and the self-developed dynamic mold control system were used for the transfer experiment of the micro-dynamic control of microstructure soft molds, including the uniaxial NPR microstructure hot-embossing replication system and the uniaxial NPR microstructure UV-curing replication system, as shown in Figure 5. 

**Figure 6:**
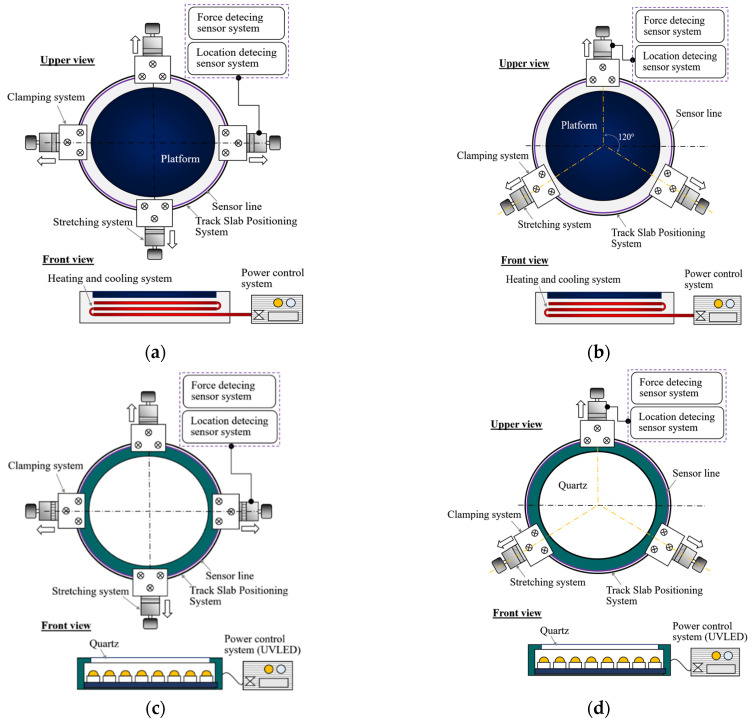
Multi-directional dynamic NPR mold clamping and P2P-forming system development (**a**) quadaxial NPR microstructure hot-embossing system; (**b**) triaxial NPR microstructure hot-embossing system; (**c**) quadaxial NPR UV-curing system; and (**d**) triaxial NPR UV-curing system.

The NPR mold clamping system for multi-directional dynamic stretching (including the quad-axial dynamic NPR mold clamping system and the triaxial dynamic NPR mold clamping system) was designed as shown in Figure 6. 

Some axial clamping systems are units and form the multi-directional dynamic mold clamping system according to the symmetry defined by precision orbit-positioning.

#### 3.2.2. Uniaxial Parallel Flexible NPR Microstructure Forming Steps 

**Figure 7:**
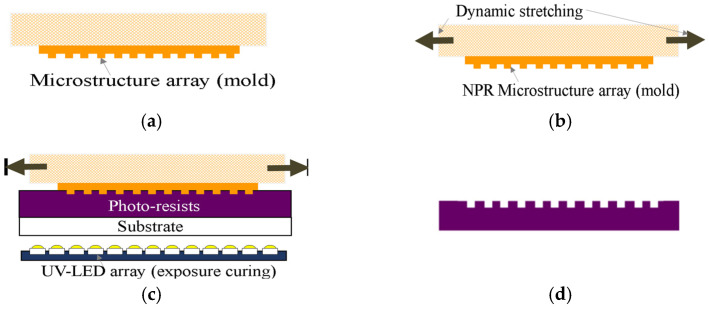
Uniaxial parallel flexible NPR microstructure mold replication steps (**a**) the clamping heads on both sides of the dynamic mold control system clamp the flexible NPR microstructure mold at the two ends and the photoresist is placed on the quartz glass substrate of the embossing and exposure system; (**b**) the non-uniformity of microstructure molds is dynamically controlled within the elastic range so that microstructures can be predicted and controlled within the elastic range; (**c**) the back-pressure is defined for embossing, exposure, and curing; and (**d**) demolding is carried out next to obtain finished products.

The uniaxial parallel NPR microstructure UV-curing replication system uses the self-developed dynamic NPR mold control system and the embossing system for embossing. The procedure is as follows: (a) the clamping heads on both sides of the dynamic mold control system clamp the flexible NPR microstructure mold at the two ends and the photoresist is placed on the quartz glass substrate of the embossing and exposure system; (b) the non-uniformity of microstructure molds is dynamically controlled within the elastic range so that microstructures can be predicted and controlled within the elastic range; (c) the back-pressure is defined for embossing, exposure, and curing; and (d) demolding is carried out next to obtain finished products, as shown in Figure 7.

## 4. Results and Discussion

### 4.1. Simulation and Analysis of NPR Structures

#### 4.1.1. Simulation and Analysis of Single SIN Waveforms and Array SIN Waveforms 

**Figure 8:**
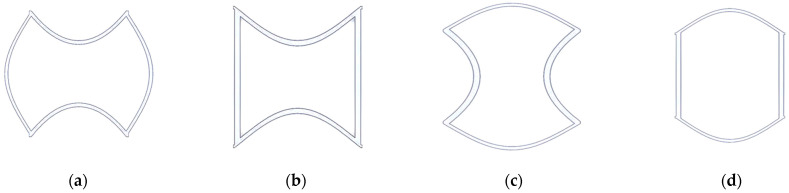
SIN waveforms: (**a**) A, (**b**) A’, (**c**) B, and (**d**) B’ types.

This study is about tunnel-shaped structures but SIN waveforms are formed during stretching. Hence, this section classifies the basic SIN waveforms into four types for preliminary simulation analysis. This study was designed based on the results of software simulation, namely A-type, A’-type, B-type, and B’-type, as shown in Figure 8. 

**Figure 9:**
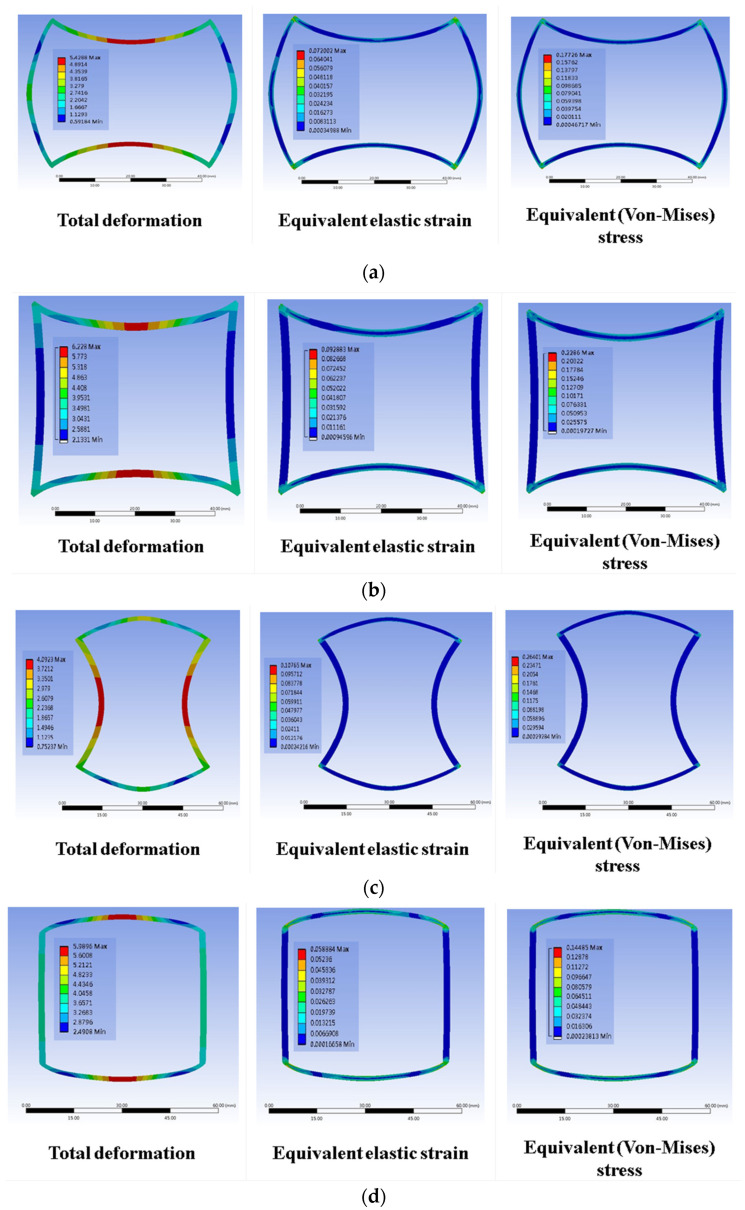
Simulation of SIN waveforms with 2.5 mm of uniaxial stretching: (**a**) A, (**b**) A’, (**c**) B, and (**d**) B’ types.

ANSYS software was used for uniaxial stretching simulation to analyze strain and geometric characteristics. The set material simulation parameters used in the simulations were as follows: density, 1.063 g/cm^3^; Young’s modulus, 2.4622 Mpa; and Poisson’s ratio, 0.5. The simulation results of the SIN waveforms with 2.5 mm of uniaxial stretching show different total deformation, strain, and stress in the four groups, as shown in Figure 9. 

**Figure 10:**
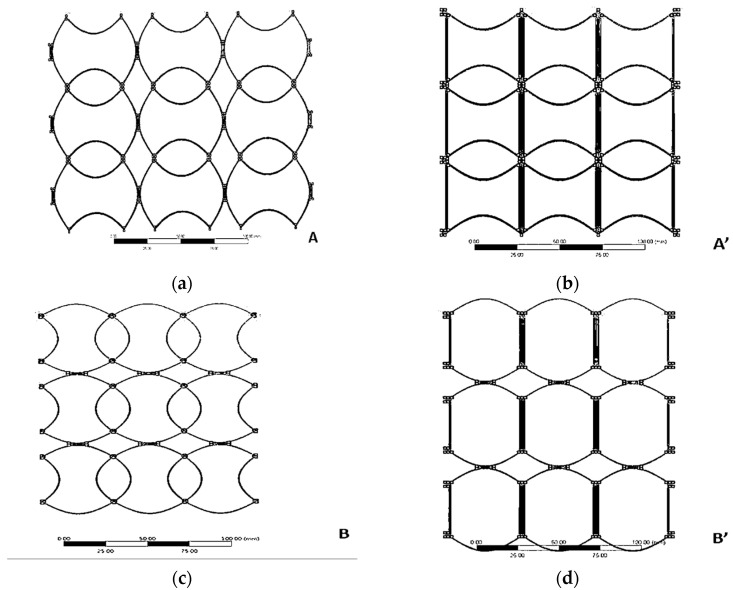
3 × 3 SIN waveforms: (**a**) A, (**b**) A’, (**c**) B, (**d**) B’, types.

In addition, they were arranged as 3 × 3 arrays (Figure 10) and 4 × 4 arrays. 

**Figure 11:**
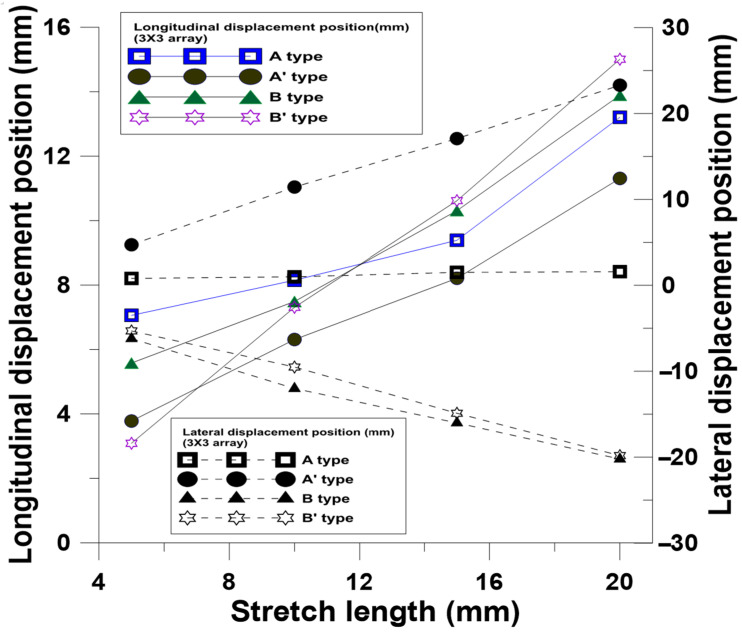
Relationship between changes in longitudinal displacement positions and lateral displacement positions of 3 × 3 NPR arrays at different stretching lengths.

**Figure 12:**
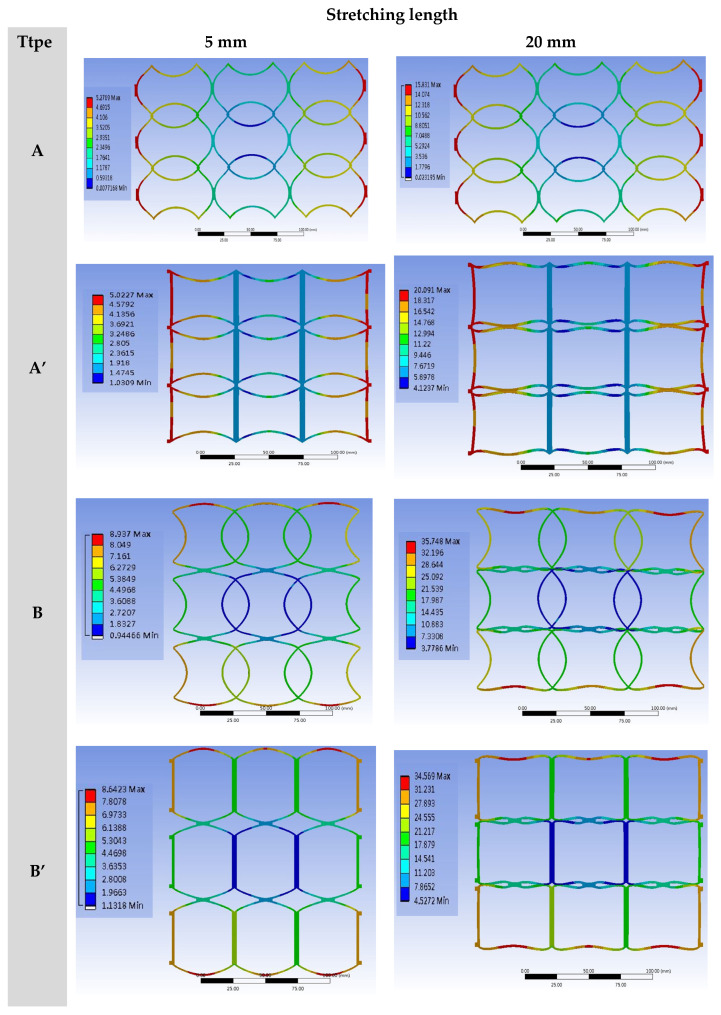
The basic SIN waveforms into four types for preliminary simula-tion analysis, and designed based on the results of software simulation, namely A-type, A’-type, B-type, and B’-type, and deformation simulation of 3 × 3 NPR arrays at different stretching lengths.

**Figure 13:**
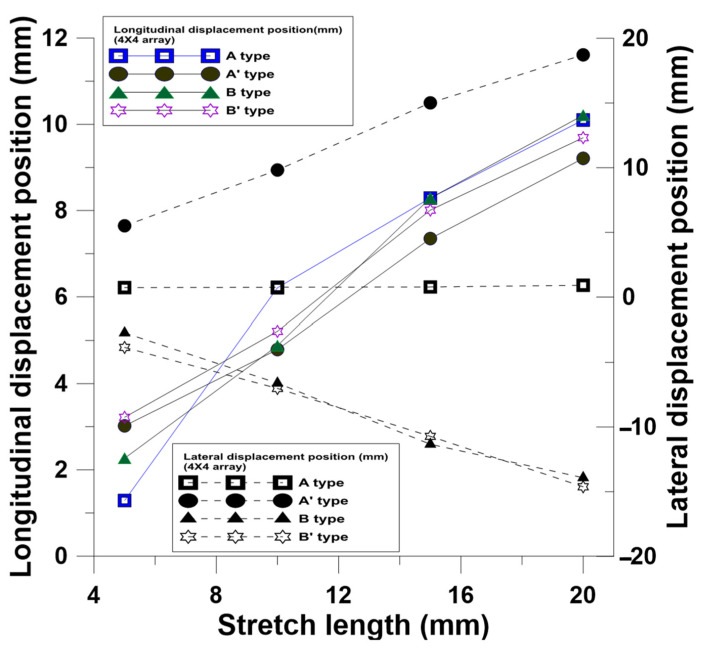
Relationship between changes in longitudinal displacement positions and lateral displacement positions of 4 × 4 NPR arrays at different stretching lengths.

**Figure 14:**
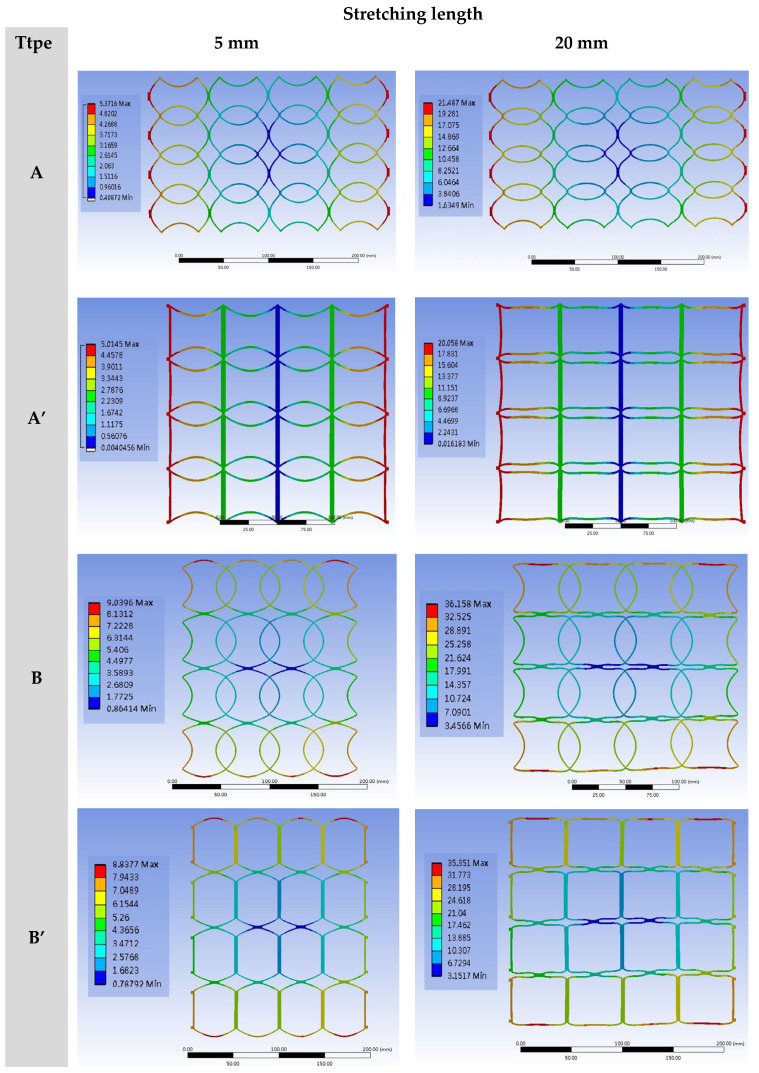
The basic SIN waveforms into four types for preliminary simula-tion analysis, and designed based on the results of software simulation, namely A-type, A’-type, B-type, and B’-type, and deformation simulation of 4 × 4 NPR arrays at different stretching lengths.

The finite element software was used for the stretching tests to observe the changes in the patterns, strains, and stresses of microstructures under different stretching conditions. The arrays (3 × 3 and 4 × 4) were stretched to 5 mm, 10 mm, 15 mm, and 20 mm by four kinds of NPR structures. According to the simulation results, the longitudinal changes of patterns with simple structures became significant with the increase in stretching. The stretching of four 3 × 3 (Figure 11 and Figure 12) and 4 × 4 (Figure 13 and Figure 14) array patterns was simulated and analyzed. The axial and radial displacements of single structures were compared and analyzed.

**Figure 15:**
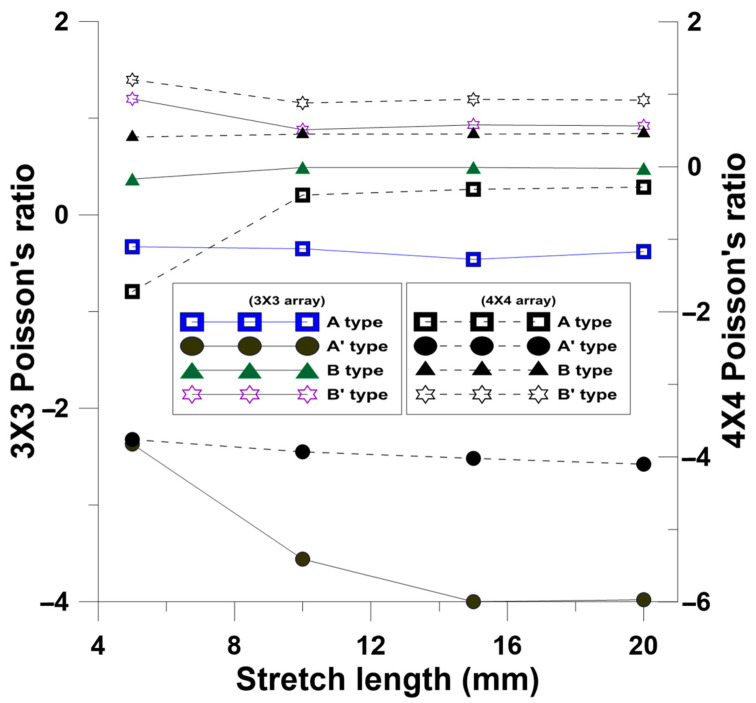
Poisson’s ratio changes of 3 × 3 and 4 × 4 NPR arrays at different stretching lengths.

The Poisson’s ratio at different stretching lengths due to axial and radial strains is shown in Figure 15. After the analysis, the Poisson’s ratios of A-type and A’-type patterns were negative, and the Poisson’s ratio of B-type and B’-type patterns were positive. In this simulation, the changes of the Poisson’s ratio became stable after they were stretched for 10 mm.

#### 4.1.2. Simulation Analysis of Stress Distribution in Molds with Different Composite Ratios and Uneven Stretching Angles 

This section discusses the simulations of the uniaxial stretching of PDMS at three different composite ratios of 5:1, 10:1, and 15:1 within the elastic range. According to the stretching test, the elastic range of PDMS at three different composite ratios is shown in Table 1.

**Figure 16:**
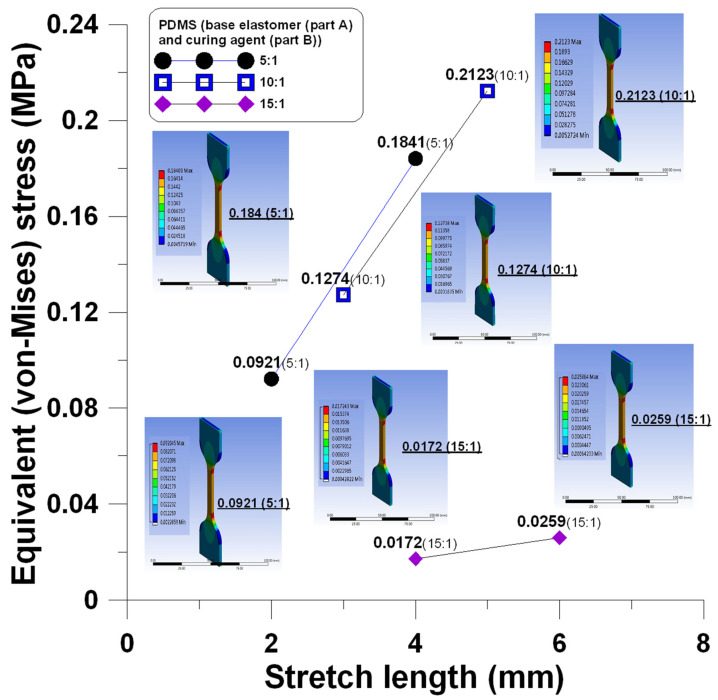
Stress changes of PDMS at a different composite ratios during stretching within the elastic range.

The simulated stress of different stretches in the elastic range (Table 2) shows that the maximum stress occured at the corners, as shown in Figure 16. 

In addition, the effects of different angles (except the horizontal angle) on the mold stress distribution were simulated. According to the simulation results, the rotation angle of PDMS at the composite ratio of 5:1 had high stress and the maximum stress would increase and become larger than that at the composite ratios of 10:1 and 15:1, as the angle would increase, as shown in Table 3.

### 4.2. Simulation and Analysis of Microstructure Mold Control by Using Parallel Dynamic NPR Molds

This section compares the stability of methods to control microstructure molds by using dynamic molds. Two parallel connections are compared in this section, namely the overall microstructure mold laying and the single-point array laying.

#### 4.2.1. Simulation Analysis of Overall Mold Laying 

The overall laying is explained in this section. According to the simulation results, in the stretching process, the warping angle increased as the stretching length increased. This method does not apply to this study.

#### 4.2.2. Simulation Analysis of Single-Point Array Laying

In the simulation analysis, the microstructure mold was 500 μm thick. Different NPR microstructure mold dimensions and thicknesses affected the warping angle perpendicular to the stretching direction at a fixed stretching length (Table 4). 

**Figure 17:**
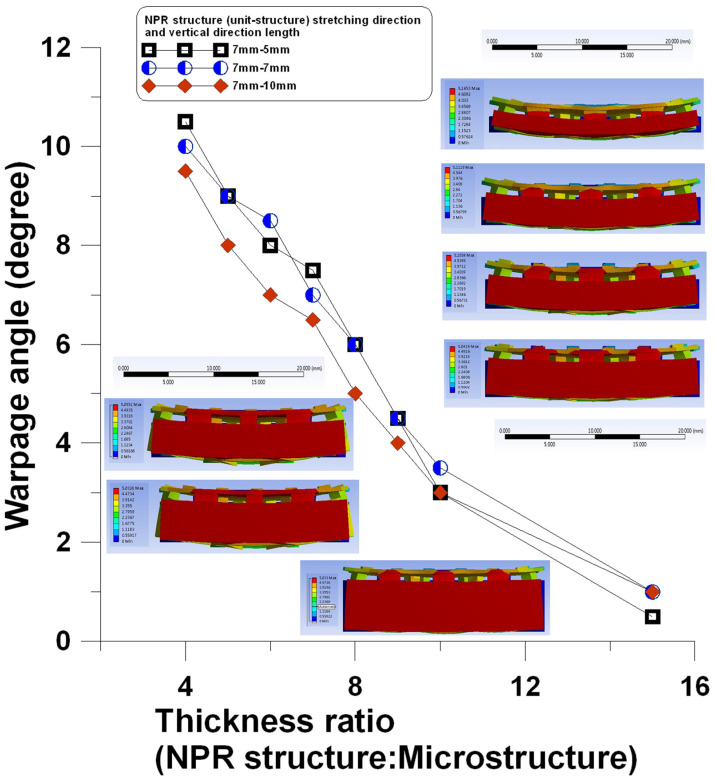
Effects of different NPR structure mold dimensions on the warping angle perpendicular to the stretching direction at a fixed stretching length.

According to the simulation results, the warping angle of the microstructure mold became smaller at a fixed stretched length as the NPR microstructure mold became thicker. A bigger single NPR structure perpendicular to the stretching direction induced the smaller average warping angle, as shown in Figure 17.

The single-point laying is explained in this section. According to the simulation results, single-point laying with (without) edge reinforcement was adopted. For stretching control, the mold with edge reinforcement could control stably and had no warping angle. The NPR effects of the dynamic mold can be reflected on the microstructure mold. The characteristic changes of the microstructure mold can be controlled indirectly through dynamic mold control.

### 4.3. Verification and Discussion of MATLAB Simulation and Dynamic NPR-Embossing Experiment

#### 4.3.1. Dynamic NPR-Embossing Replication Predicted by MATLAB Simulation 

**Figure 18:**
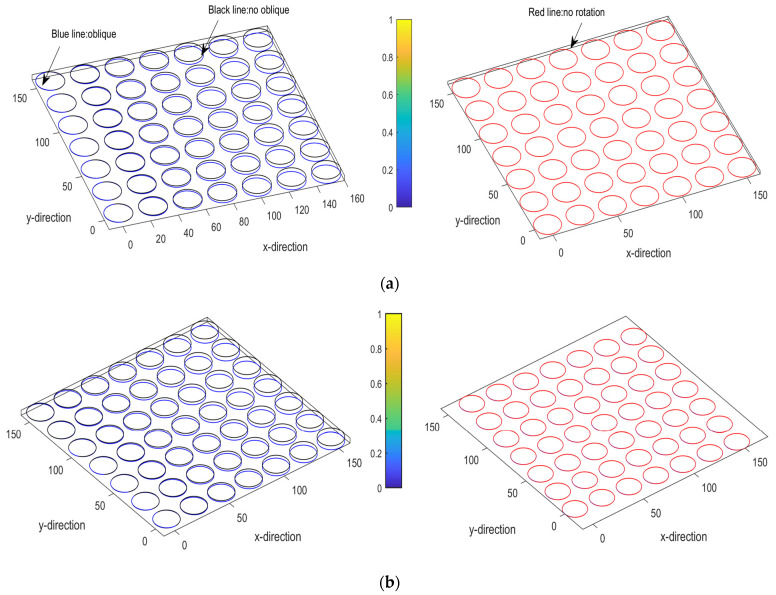
Dynamic NPR-embossing replication of 7 × 7 arrayed micro-column structures were simulated and predicted by MATLAB: (**a**) an inclination angle of 1 degree in uniaxial unstable stretching and (**b**) embossing replication prediction of an inclination angle of 1 degree and a rotation angle of 5 degrees in multi-directional dynamic stretching.

MATLAB is used to predict and analyze the embossing replication of array microstructures after dynamic NPR-stretching. This section predicted stable stretching and embossing of 7 × 7 arrayed microcolumn structures by MATLAB in uniaxial quantitative stretching. It then compared the forming at an inclination angle of 1 degree in uniaxial unstable stretching, as shown in Figure 18a. In multi-directional dynamic stretching, the molds lose their original angle due to external forces. They are rotated to a certain degree due to the external thrust. It was predicted that, at the inclination angle of 1 degree and at the additional rotation angle of 5 degrees (Figure 18b), it is enough to understand the errors and interpretations, which may be caused by the dynamic stretching and through the simulation prediction.

#### 4.3.2. Discussion of Uniaxial Dynamic NPR Microstructure Replication

**Figure 19:**
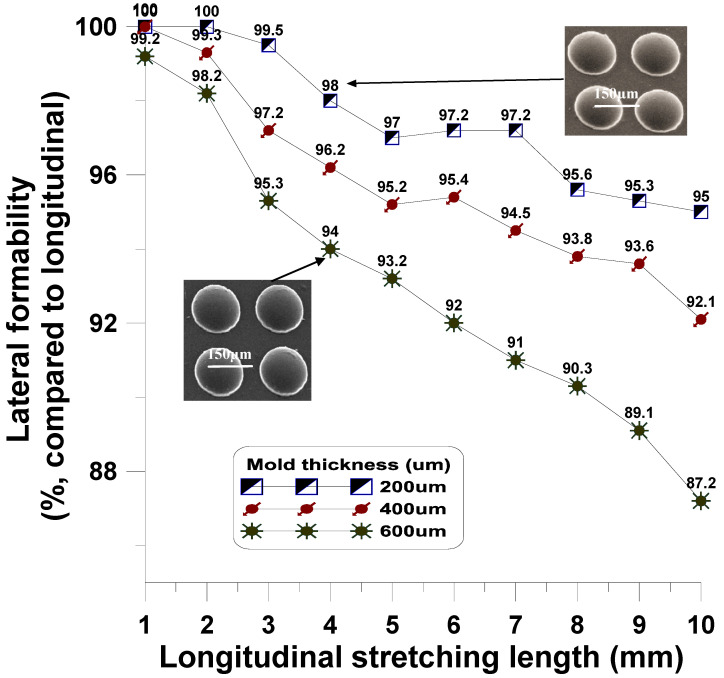
Formability of microstructure molds with different thicknesses in the uniaxial dynamic NPR microstructure mold replication.

The uniaxial dynamic stretching NPR structure is used to control the embossing replication of microstructure (diameter 150 µm, height 75 µm) array molds within the elastic range. According to the experimental results, the formability of P2P hot-embossed and P2P UV-cured (pressurized to 0.16 Mpa) materials is close to that of PC and photoresist-formed materials. However, as the thickness of microstructure-molds changed (200 µm, 400 µm, and 600 µm), the control of the radial stretching was insufficient for embossing replication, as shown in Figure 19. 

#### 4.3.3. Discussion of Multi-Directional Dynamic NPR Microstructure Replication

The multi-directional dynamic stretching NPR structure is used to control the embossing replication of microstructure (diameter 150 µm, height 75 µm) array molds within the elastic range. According to the experimental results, the formability of P2P hot-embossed and P2P UV-cured (pressurized to 0.16 Mpa) materials is close to that of PC and photoresist-formed materials. The three-directional and four-directional radial stretching of the system can be controlled. The changes in the thickness of microstructure molds and the increase in the length after stretching had no effects on the actual formability.

In this experiment, we used uniform synthesis parameters (10:1) and the same curing temperature (140 °C), therefore the molds share the same Young’s modulus values, while the mold with NPR geometric position changes and the stress distribution (Young’s modulus) of molds in dynamic stretching were changed. This was to adjust for the different research requirements of NPR structures and to build a mechanical model of the flexible NPR microstructure molds.

## 5. Conclusions

This study is committed to developing a dynamic forming mechanism and forming process of negative Poisson’s ratio elastomer molds, as well as is committed to the development and innovation of this process. This study integrates properties of elastomer and micro-electro–mechanical key technologies through systematic research. In addition, it innovatively develops adjustable negative Poisson’s ratio microstructures. The controllable characteristics of elastomer was used to achieve low cost and fast control. The control characteristics of NPR structure molds were analyzed through a series of simulation analyses. The microstructures were verified, replicated, and transferred for comparison. A uniaxial parallel flexible NPR microstructure-mold fabrication method was proposed and a series of dynamic NPR P2P-forming systems were designed. According to the experiment, the multi-directional dynamic NPR microstructure replication process for the dynamic forming technology of negative Poisson’s ratio elastomer molds is good and can achieve stable control.

## Figures and Tables

**Table 1:** Stretching test to test the elastic range of PDMS at three different ratios.

Elastic Range (mm) (Experiment)	PDMS (Base Elastomer (Part A) and Curing Agent (Part B))		
	5:1	10:1	15:1
1	4.55	5.88	7.21
2	4.59	5.84	7.13
3	4.62	5.87	7.18
Average	4.59	5.86	7.17

**Table 2:** Effects of stretching length on the mold stress within the elastic range of PDMS at different composite ratios.

Stretch Length (mm)	PDMS (Base Elastomer (Part A) and Curing Agent (Part B))		
	5:1	10:1	15:1
	Equivalent (Von-Mises) Stress (MPa)		
2	0.0921	-	-
3	-	0.1274	-
4	0.1841	-	0.0172
5	-	0.2123	-
6	-	-	0.0259
Equivalent stress increase per millimeter (MPa)	0.0463	0.0425	0.0044

**Table 3:** Effects of rotation angles on the maximum mold stress within the elastic range of PDMS at different composite ratios.

Rotation Angle (Degree)	PDMS (Base Elastomer (Part A) and Curing Agent (Part B))		
	5:1	10:1	15:1
	Equivalent (Von-Mises) Stress (MPa)		
15	0.1848	0.1201	0.0258
25	0.1865	0.1204	0.0260
Equivalent stress increase per degree (KPa)	0.17	0.03	0.02

**Table 4:** Simulation results of the warping angle perpendicular to the stretching direction at a fixed stretching length with different NPR structure mold dimensions.

Thickness Ratio (Microstructure Mold:NPR Structure Mold)	NPR Structure (Unit-Structure), Stretching Direction, and Vertical Direction Length		
	7 mm, 5 mm	7 mm, 7 mm	7 mm, 10 mm
	Warping Angle (Degree)		
1:4	10.5	10	9.5
1:5	9	9	8
1:6	8	8.5	7
1:7	7.5	7	6.5
1:8	6	6	5
1:9	4.5	4.5	4
1:10	3	3.5	3
1:15	0.5	1	1

## Data Availability

Not applicable.
